# BONCAT-FACS-Seq reveals the active fraction of a biocrust community undergoing a wet-up event

**DOI:** 10.3389/fmicb.2023.1176751

**Published:** 2023-06-26

**Authors:** Ryan V. Trexler, Marc W. Van Goethem, Danielle Goudeau, Nandita Nath, Rex R. Malmstrom, Trent R. Northen, Estelle Couradeau

**Affiliations:** ^1^Intercollege Graduate Degree Program in Ecology, Huck Institutes of the Life Sciences, The Pennsylvania State University, University Park, PA, United States; ^2^Environmental Genomics and Systems Biology Division, Lawrence Berkeley National Laboratory, Berkeley, CA, United States; ^3^Biological and Environmental Sciences and Engineering Division, King Abdullah University of Science and Technology, Thuwal, Saudi Arabia; ^4^Lawrence Berkeley National Laboratory, DOE Joint Genome Institute, Berkeley, CA, United States; ^5^Department of Ecosystem Science and Management, The Pennsylvania State University, University Park, PA, United States

**Keywords:** BONCAT, biocrust, soil metagenomics, active microorganisms, soil wetting

## Abstract

Determining which microorganisms are active within soil communities remains a major technical endeavor in microbial ecology research. One promising method to accomplish this is coupling bioorthogonal non-canonical amino acid tagging (BONCAT) with fluorescence activated cell sorting (FACS) which sorts cells based on whether or not they are producing new proteins. Combined with shotgun metagenomic sequencing (Seq), we apply this method to profile the diversity and potential functional capabilities of both active and inactive microorganisms in a biocrust community after being resuscitated by a simulated rain event. We find that BONCAT-FACS-Seq is capable of discerning the pools of active and inactive microorganisms, especially within hours of applying the BONCAT probe. The active and inactive components of the biocrust community differed in species richness and composition at both 4 and 21 h after the wetting event. The active fraction of the biocrust community is marked by taxa commonly observed in other biocrust communities, many of which play important roles in species interactions and nutrient transformations. Among these, 11 families within the Firmicutes are enriched in the active fraction, supporting previous reports indicating that the Firmicutes are key early responders to biocrust wetting. We highlight the apparent inactivity of many Actinobacteria and Proteobacteria through 21 h after wetting, and note that members of the Chitinophagaceae, enriched in the active fraction, may play important ecological roles following wetting. Based on the enrichment of COGs in the active fraction, predation by phage and other bacterial members, as well as scavenging and recycling of labile nutrients, appear to be important ecological processes soon after wetting. To our knowledge, this is the first time BONCAT-FACS-Seq has been applied to biocrust samples, and therefore we discuss the potential advantages and shortcomings of coupling metagenomics to BONCAT to intact soil communities such as biocrust. In all, by pairing BONCAT-FACS and metagenomics, we are capable of highlighting the taxa and potential functions that typifies the microbes actively responding to a rain event.

## Introduction

Biological soil crusts (biocrusts) are an assemblage of organisms that form a perennial, well-organized surface layer in soils (Weber et al., 2022; Garcia-Pichel, 2023). Covering 12% of Earth’s terrestrial surface (Rodriguez-Caballero et al., 2018), biocrusts are the dominant land cover in arid and semi-arid environments where they mediate key ecological processes and contribute a multitude of essential ecosystem services (Rodríguez-Caballero et al., 2018). Carbon-fixing Cyanobacteria within early successional biocrusts are the dominant primary producers in these soils and add substantial carbon to the soil carbon pool (Chen et al., 2014; Büdel et al., 2018). Similarly, nitrogen-fixing organisms supply fixed nitrogen to the soil which improves soil fertility and productivity (Elbert et al., 2012; Barger et al., 2016; Ferrenberg et al., 2018). Additionally, biocrust organisms also produce extracellular polysaccharides (EPS) that stabilize the soil surface (Mazor et al., 1996), prevent soil erosion (Belnap and Gillette, 1998), promote soil aggregation (Belnap and Gardner, 1993), and regulate soil hydrology (Belnap, 2006).

Although essential to global biogeochemical cycling and provisioning ecosystem services in arid lands, most biocrust organisms remain dormant during long periods of soil desiccation where their activity is largely dependent on moisture inputs from sporadic and often brief rainfall events. This soil wetting triggers a time-dependent response by the biocrust microbial community whereby the composition (Angel and Conrad, 2013; Karaoz et al., 2018; Van Goethem et al., 2019; Baubin et al., 2022), transcriptional patterns (Rajeev et al., 2013), and metabolic output (Swenson et al., 2018) of the community shifts within hours and days after wetting. The dominant cyanobacterial member in early successional biocrusts, *Microcoleus* sp., is immediately resuscitated and initiates cellular metabolism and photosynthesis (Rajeev et al., 2013). *M. vaginatus* is known to symbiotically exchange carbon (C) and nitrogen (N) with heterotrophic nitrogen fixers that are co-localized within its’ bundle sheath (Nelson et al., 2020), though it remains unknown how quickly these symbiotic diazotrophs respond to soil wetting in relation to *Microcoleus* species. Members of the Firmicutes – *Alicyclobacillaceae*, *Bacillaceae*, and *Planococcaceae* – increase in abundance significantly within 18 h of soil wetting (Karaoz et al., 2018), and subsequently decline rapidly due to predation by *Caudovirales* phages (Van Goethem et al., 2019). These studies provide evidence of dynamic and complex responses to soil wetting; however, with the exception of *M. vaginatus*, it remains unknown which members activate quickly in response to available water (e.g., within a few hours) and which taxa remain dormant within the community. Additionally, the metabolic capabilities and potential nutrient cycling capacities of these early responders is not known. An understanding of which organisms actively respond to a wetting event, and what functions these organisms perform, is needed to fill these gaps and to provide a framework for explaining the ecological processes and nutrient cycling occurring in biocrust ecosystems.

Identifying the active microorganisms in complex environments has recently garnered significant attention. A variety of methods have been developed to accomplish this, including stable isotope probing (SIP), bromodeoxyuridine (BrdU) labeling, and bioorthogonal non-canonical amino acid tagging (BONCAT). While SIP and BrdU both require cells to be actively reproducing (i.e., undergoing DNA replication) in order to detect activity, BONCAT identifies organisms that are actively producing new proteins, regardless of whether or not they are replicating DNA. Moreover, mRNA-based studies (e.g., transcriptomics) can be used to identify functional activity; however, mRNA abundance is commonly not synchronous with protein abundance (Vélez-Bermúdez and Schmidt, 2014; Fukao, 2015) which is better representative of cellular activity. For example, even dormant cells contain mRNA (Setlow and Christie, 2020), which if the taxonomy of these transcripts were assigned in a metatranscriptomic study would suggest these taxa were functionally active. Furthermore, taxonomic assignment from transcipts offers less taxonomic resolution than from marker genes (e.g., 16S rRNA or Internally Transcribed Spacer regions), which can be paired with BONCAT-FACs probing.

BONCAT reveals cellular activity by using non-canonical amino acids [e.g., homopropargylglycine (HPG)] that are imported by microorganisms and incorporated into newly made proteins during translation from mRNA. Because of this, BONCAT distinguishes cells so that the resulting proteins can be tagged with a fluorescent marker in a click chemistry reaction to probe the activity of the cell. BONCAT has been integrated with fluorescence activated cell sorting (FACS) which allows for the collection of pools of cells that are viable as well as either active or inactive based on the fluorescence from the “clicked” proteins. This method (BONCAT-FACS) has been paired with marker gene sequencing in diverse ecosystems (Hatzenpichler et al., 2016; Reichart et al., 2020; Valentini et al., 2020; Du and Behrens, 2021; Taguer et al., 2021), including bulk soil (Couradeau et al., 2019b), to investigate the activity of microorganisms. We believe BONCAT-FACS is well suited to study the activity of biocrust communities because a simulated rain event can easily be used to add the required non-canonical amino acid (e.g., HPG).

In this study, we empirically identified the taxa within a biocrust community that responded to a simulated rain event and characterized the functional potential of these taxa by coupling BONCAT-FACS with shotgun metagenomic sequencing (Seq). Here, BONCAT-FACS-Seq was applied to three biological replicates of an early successional biocrust and destructively sampled at 4 and 21 hrs after a wetting event. To our knowledge, this research represents the first time BONCAT-FACS-Seq was applied to biological soil crusts. Using this novel methodology, we find that a simulated rain event activates a select fraction the biocrust microbial community which includes taxa that have both previously been found to play important roles in biocrust communities as well as taxa that have remained relatively undescribed in biocrusts. Functions related to defense against predation, as well as nutrient recycling and scavenging, are enriched in the active metagenomes compared to inactive metagenomes, highlighting the potential importance of these ecological processes within hours after biocrust wetting.

## Materials and methods

### Biocrust field sampling, microcosm initiation, and microcosm sampling

Three replicates of biocrust were sampled from the Colorado Plateau near Moab, Utah (GPS coordinates: 38.715278, −109.692500). Undisturbed, early successional (Cyanobacteria-dominated) biocrusts located away from vegetation were selectively targeted for sampling. Biocrust samples were taken in fall 2014 following a natural rain event that wet the soil sufficiently to sample as described previously (Van Goethem et al., 2019). Specifically, the samples of biocrust were collected by pressing 60 mm × 15 mm Petri plates into the soil and a sterile spatula was used to cut horizontally under the plate to remove the intact biocrust. Excess soil that overflowed the plate was carefully scraped from the sample. Samples were allowed to fully dry upside down the day before being packed for transportation to the DOE Joint Genome Institute, California (JGI). They were stored in the dark at <20% relative humidity until being revived via the addition of water for the experiment in spring 2017.

**FIGURE 1:**
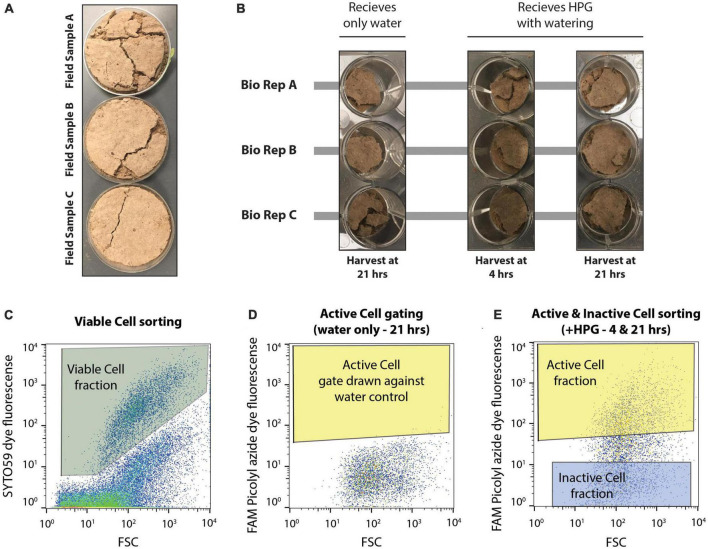
Experimental set up of microcosms and design of flow cytometry-based cell sorting. **(A)** Three biological replicate field samples of biocrusts were collected from Moab, UT in petri dishes. **(B)** Field samples were distributed to a total of nine wells of a 12 well plate. Each biological replicate (bio rep) was distributed to three wells each. This first set of biocrusts received only water during the simulated rain event and was harvested 21 hrs later. The remaining six wells received HPG during soil wetting and were harvested at either 4 or 21 hrs after incubation. **(C)** SYTO59 fluorescence was used to collect intact, viable cells (Viable Cell fraction), discriminating these particles from non-stained particles. **(D)** Microcosms receiving only water were used to identify the background FAM Picolyl azide dye fluorescence. Fluorescence above this background (giving a false positive rate of >0.05%) represented active cells (Active Cell gate) and any particles falling within both the Viable Cell gate and the Active Cell gate were assigned to the Active Cell fraction. **(E)** The Inactive Cell gate was drawn at least a half an order of magnitude lower in FAM Picolyl azide dye fluorescence, and cells within the Viable Cell gate and Inactive Cell gate were assigned to the Inactive Cell fraction when sorting, while cells falling within the Viable Cell and Active Cell gate were assigned to the Active Cell fraction. Cell sorting was performed on microcosms receiving HPG and harvested at either 4 or 21 hrs following the wetting event.

For this experiment, microcosms of biocrust were prepared in a 12-well plate. The three biological replicates of biocrust sample from the field were each aseptically subsampled into three separate wells for each biological replicates, resulting in nine total microcosms with roughly 2–3 g of biocrust material (Figure 1 and Supplementary Figure 1). One well per biological replicate (*n* = 3 wells) were assigned as water controls and were harvested at 21 hrs after wetting. The remaining six wells (two microcosms per biological replicate) were designated to either be destructively sampled at 4 or 21 hrs following wetting. This allowed for destructive harvesting of each of the three biological replicates at two timepoints (4 hrs after wetting and 21 hrs after wetting) and one water control per biological replicate harvested at 21 hrs after wetting. All nine microcosms were wet with a simulated 3 mm rain event. Here, the three water control microcosms received 600 μl of sterile deionized water, while the other six microcosms each received 600 μl of a sterile 50 μM L-homopropargylglycine (HPG) solution (Click Chemistry Tools; Scottsdale, AZ, USA). HPG is a methionine analog containing a terminal alkyne that allows downstream click chemistry to label newly synthesized proteins (Hatzenpichler and Orphan, 2015; Couradeau et al., 2019b).

Once re-wetted, the microcosms were incubated at 23°C and received 100 μmol of photons m^–2^ s^–1^ for 12 hrs before incubating in the dark for the remaining 9 h. The microcosms were destructively harvested at either 4 or 21 hrs after wetting (water controls were harvested at 21 h). At harvest, the microcosms were aseptically transferred from the 12-well plate to 15 ml conical tubes containing 800 μl of EDTA (500 mM, pH 8) and a mixture of 8 ml of a PBS (1X)/Tween20 (0.02%). The samples were vortexed for 5 min and centrifuged at 500 G for 5 min before collecting the supernatant. Vortexing, centrifugation, and supernatant collection was repeated two additional times, and in total 700 μl of supernatant was collected from each microcosm. The cell suspensions were mixed with 350 μl of 20% glycerol (10% glycerol final concentration) and stored at −20°C for downstream processing.

The click reaction was performed as described before (Couradeau et al., 2019b). All reagents for click reactions below were purchased from Click Chemistry Tools (Click Chemistry Tools; Scottsdale, AZ, USA). Here, while the frozen cell suspensions were thawing at 4°C for 1 h, the click-reaction mixture was prepared by first incubating the dye premix (100 μM copper sulfate, 500 μM tris-hydroxypropyltriazolylmethylamine, 5 μM FAM picolyl azide dye) for 3 min in the dark before being mixed with the reaction buffer (5 mM sodium ascorbate and 5mM aminoguanidine HCl in 1X PBS). Once the cell suspensions were thawed, the cells were collected on a 0.2 μm GTTP isopore 25 mm diameter filter (MilliporeSigma; Burlington, MA, USA) and rinsed with 7 ml of 1X PBS. The click reaction mixture was added to the collected cells by placing the filters on a glass slide, adding 80 μL of the click reaction mixture, and covering with a coverslip. The slides were incubated in the dark for 30 min before being placed in three successive baths of 20 ml of 1X PBS for 5 min each. The filters were removed from the slides and transferred to 5 ml tubes (BD-Falcon 5 ml round bottom tube with snap cap, Corning Inc., Corning, NY, USA) that contained 2 ml of 0.02% Tween^®^ 20 in PBS. The filters were then vortexed at maximum speed for 5 min to detach the cells from the filter. The cells were incubated for 20 min at 25°C before being stored at 4°C until cell sorting.

Flow cytometry based cell sorting of the collected, clicked extracts was performed as described before (Couradeau et al., 2019b) and the data regarding cell sorting for this experiment can be found in Supplementary Figure 2. All clicked cell samples were first counter-stained with SYTO59 (Invitrogen, Eugene OR, USA). DNA dye by incubating for 5 min at room temperature (5 μM final concentration of SYTO59). This allows for the separation of intact cells from background soil particles, as SYTO59 is a general DNA staining dye. Cells were then filtered through a 35 μm filter (BD-falcon 5 ml tube with cell strainer cap, Corning Inc., Corning, NY, USA) before being loaded on the cell sorter (BD-Influx, BD Biosciences; San Jose, CA, USA). The cell sorting was performed using a nested gate strategy. SYTO59-stained cells were gated against unstained cells using the red channel (SYTO59, excitation: 640 nm, emission: 655–685 nm). Three technical replicates (100,000 events each) were collected from this gate for all the samples – cells from this gate are called “Viable Cell” fractions. The BONCAT gate was set as a nested gate on the green channel (FAM Picolyl dye, excitation: 488 nm, emission: 530 nm) under the SYTO59 gate, therefore visualizing the active microbes among cells only. The BONCAT positive gate (“Active Cell” fraction) was drawn against two Water Control samples (i.e., incubated without HPG) at each time point, controlling for a false positive discovery rate of <0.31%. The BONCAT negative gate (Inactive Cells) was drawn at low fluorescence levels, at least half a log scale lower from the bottom of the BONCAT positive gate (see Figure 1 and Supplementary Figure 2 for details about the gating strategy). In total, 100,000 events were collected from the Active Cell and Inactive Cell gates for each biological replicate at each timepoint. Unfortunately, the amount of sample did not allow us to collect technical replicates for these fractions. All sorted cell fractions were kept at −80°C until further processing.

### DNA extraction and metagenomic sequencing

A total of 33 samples were extracted for metagenomic DNA sequencing (Supplementary Figure 2). This included the following sets of samples: (1) A subsample from each of the three dry biocrust replicates before microcosm preparation (“Dry Biocrust,” one metagenome × three biological replicates, *n* = 3); (2) Subsamples of SYTO-stained cell fractions collected prior to BONCAT cell sorting (“Viable Cells,” one metagenome × three technical replicates × three biological replicates × two timepoints, *n* = 18); (3) SYTO-stained cell fractions that fell outside the BONCAT fluorescent label gating (“Inactive Cells,” one metagenome × three biological replicates × two timepoints, *n* = 6); (4) SYTO-stained cell fractions that fell inside the BONCAT fluorescent label gating (“Active Cells,” one metagenome × three biological replicates × two timepoints, *n* = 6).

DNA extractions from the Dry Biocrust samples were prepared using the PowerSoil DNA extraction kit (Qiagen; Hilden, Germany) with the following modifications. Cell lysis was performed using the TissueLyser II (Qiagen; Hilden, Germany) for 10 min at 30 Hz, and a total of 800 μl of lysate was collected. Further, 850 μl of supernatant was collected and processed after washing with solution CD2 and CD3. The solution C5 wash was modified to a volume of 1000 μl. Elution was performed using 100 μl of TE buffer (pH 8.2) and incubating at room temperature for 2 min before centrifuging at 10,000 G.

Cell fraction samples collected from cell sorting (Viable Cells, Inactive Cells, Active Cells) were extracted using the prepGEM (MicroGEM International PLC; Southampton, UK) chemical lysis as follows. Cell samples were first centrifuged at 3,800 G for 1 hrs at 10°C to pellet the collected cells and then centrifuged upside down briefly for 10 s to remove the supernatant. Following supernatant removal, 2 μl of prepGEM mix was added to each cell pellet (final volume per pelleted sample: 0.2 μl buffer, 0.025 μl prepGEM reagent, 0.025 μl lysozyme, 1.75 μl water). Cell fractions were lysed at 37°C for 30 min and subsequently 75°C for 40 min. DNA extracts were stored at −20°C until processing for sequencing.

DNA extracts were prepared for metagenomic sequencing using the Nextera XT Library Prep kit (Illumina; San Diego, CA, USA) following the manufacturer’s recommendations. Following tagmentation, Dry Biocrust samples were amplified with 12 cycles of PCR while those from the Viable Cells, Inactive Cells, and Active Cells fractions were amplified with 25 cycles of PCR. The resulting libraries were cleaned using the Agencourt AMpure XP kit (Beckman Coulter; Brea, CA) and assessed on the Agilent BioAnalyzer 2100 (Agilent Technologies; Santa Clara, CA, USA).

Sequencing for the Dry Biocrust samples was performed on an Illumna NovaSeq (2 × 150 bp chemistry) while the remaining cell fractions were sequenced on an Illumina NextSeq instrument (2 × 150 bp chemistry). All sequencing was performed at JGI according to their standard workflow. In total, metagenomic sequencing provided a total of ∼170 Gbp in 1,128,245,224 raw sequences across the 33 biocrust samples. We estimated our sequence coverage for each metagenome (Supplementary Figure 3) using Non-pareil v3.30 which relies on read redundancy to calculate sequencing depth (Rodriguez-R et al., 2018). The raw sequences were filtered using RQCFilter to trim adapters, filter artifacts and contaminants and to cull low quality reads, which was followed by read merging with bbmerge (Bushnell et al., 2017). Raw sequences from the 33 metagenomes were submitted to the NCBI SRA under the accession PRJNA938738.

### Bioinformatic and statistical analyses

Taxonomic assignments of quality-control filtered reads were made using kraken2 (Wood et al., 2019) and taxonomic abundances were estimated using bracken (Lu et al., 2017). The resulting taxa abundance table was analyzed using the R (v4.2.2) statistical software (R Core Team, 2022a). Taxa relative abundances were visualized using the R packages “phyloseq” (McMurdie and Holmes, 2013) and “microshades” (Dahl et al., 2022). The “vegan” package (Oksanen et al., 2008) was used to estimate alpha diversity metrics, calculate Bray-Curtis dissimilarities, and create non-metric multidimensional scaling (NMDS) plots. Normality of response variables were checked via Shapiro–Wilk tests (α = 0.05) and Q-Q plots with the “stats” package. ANOVA and Tukey’s HSD *post-hoc* tests (α = 0.05) were performed using the R package “stats” (R Core Team, 2022b) with taxa richness, Shannon diversity, and Pielou’s evenness as response variables. *Post hoc* p-values were corrected for multiple comparisons using the Benjamini-Hochburg adjustment (Benjamini and Hochberg, 1995). PERMANOVA analyses (α = 0.05) were performed on the Bray-Curtis dissimilarities using the “vegan” package. Within-group beta-dispersion estimates were calculated in R using Euclidian distances to group centroids in the NMDS space and tested using a two-way ANOVA and Tukey’s HSD tests (α = 0.05). Similar to the alpha diversity measures above, ANOVA and Tukey’s HSD tests with multiple comparisons corrections were performed on the beta-dispersion estimates. “DESeq2” (Love et al., 2014) was used to determine differentially abundant taxa between the Inactive Cell and Active Cell fractions at 4 and 21 h. DESeq2 was run on the data summarized at the species, genus, family, and phylum levels in order to determine discriminatory taxa at each level.

In order to probe potential functional capabilities, metagenomes were assembled and annotated via the Integrated Microbial Genomes and Metagenomes (IMG/M). All cell fractions (Viable Cell, Active Cell, Inactive Cell) were annotated using the IMG/M Annotation Pipeline v4 pipeline (Markowitz et al., 2014) while the three Dry Biocrust metagenomes were annotated via the IMG/M Annotation Pipeline v5 pipeline (Chen et al., 2019). The resulting assembled and annotated metagenomes used in this study (Supplementary Table 1) can be found via JGI Gold^1^ under the Gold Study ID Gs0131328 and additional data regarding these metagenomes can be found in Supplementary Table 2. Samples SYTO_8 (Viable Cell fraction, Biological Replicate C, Technical Replicate 2, 21 hrs post-wetting) and SYTO_18 (Viable Cell fraction, Biological Replicate C, Technical Replicate 2, 4 hrs post-wetting) were not available from IMG/M because of incomplete assembly with the IMGv4 assembly and annotation pipeline. From the remaining 31 assembled metagenomes, Clusters of Orthologous Genes (COGs) were downloaded from IMG/M for analysis with the R Statistical software. The “vegan” package was used to calculate Bray-Curtis dissimilarities on the COG abundance table and to create NMDS ordination plots. PERMANOVA and beta-dispersion analyses (α = 0.05) were performed as described above for the taxonomic data. “DESeq2” was used to determine differentially abundant COGs between the Inactive and Active Cell fractions at 4 and 21 h. DESeq2 was run on the data summarized at both the COG, “COG Category,” and “COG Pathway” levels. All plots were generated using “ggplot2” package (Wickham, 2016) and further refined using Adobe Illustrator v25.4.1.

## Results

In total, we sequenced 33 metagenomes (Supplementary Figure 2), yielding ∼170 Gbp in sequence data. Among these, a metagenome was generated from each of the three biological replicates of Dry Biocrust samples (*n* = 3), and the remaining 30 metagenomes were prepared from sorted cell fractions that were derived from the three biological replicates of biocrust at two timepoints following a simulated rain event (4 and 21 h) and by applying these samples to BONCAT-FACS. The sorted cell fractions consisted of three populations of cells (Viable Cell, Active Cell, and Inactive Cell). The Active Cell and Inactive Cell fractions each had a metagenome that were generated from the three biological replicates across two sampling timepoints (*n* = 12 in total: two fractions × three biological replicates × two timepoints), while the Viable Cell fraction had three technical replicate metagenomes per biological replicate at each of the two timepoints (*n* = 18 in total: one fraction × three biological replicates × three technical replicates × two timepoints).

### Metagenome diversity and taxonomic composition

**FIGURE 2:**
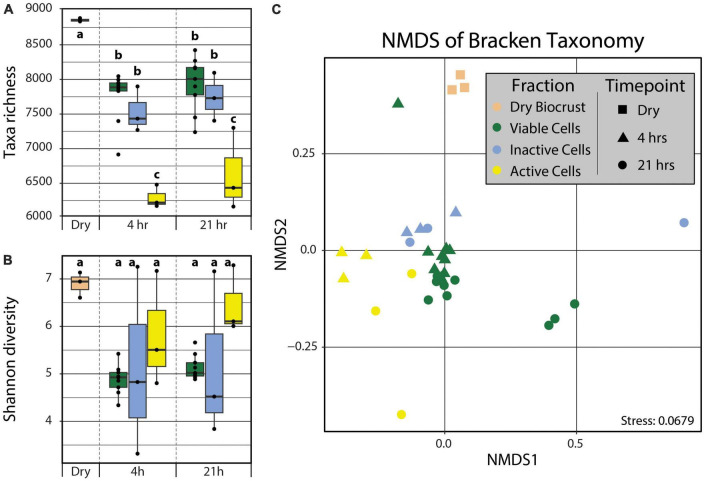
Taxa richness **(A)** and Shannon diversity **(B)** calculated from kraken2/bracken output. Differences in taxa richnesss and Shannon diversity among groups (“abc”) were tested using two-way ANOVAs with *post hoc* Tukey’s HSD tests (α = 0.05). No statistical differences were observed for Shannon diversity. **(C)** Non-metric multidimensional scaling of the kraken2/bracken taxonomy using Bray–Curtis distances. PERMANOVA analyses demonstrated significant grouping by Fraction (Pr(>F) = 0.0009). Boxplot and point color indicates Fraction (tan – Dry Biocrust; green – Viable Cells; blue – Inactive Cells; yellow – Active Cells) and point shape specifies Timepoint (square – Dry Biocrust; triangle – 4 hrs; circle – 21 hrs).

In order to detect the taxonomic differences between the active and inactive components of a biocrust community following a simulated rain event, the metagenomes were analyzed using kraken2 (Wood et al., 2019) and bracken (Lu et al., 2017). We observed significant differences in the taxonomic diversity and composition across both time (i.e., 4 and 21 hrs post-wetting) and cell fractions (i.e., Active Cell and Inactive Cell populations). The Dry Biocrust metagenomes showed the highest taxa richness (8357 ± 14), followed by the Viable Cell (4 h: 7255 ± 122, 21 h: 7433 ± 129) and Inactive Cell (4 h: 7034 ± 188, 21 h: 7243 ± 200) fraction metagenomes, while the Active Cell (4 h: 5791 ± 95, 21 h: 6133 ± 343) fraction metagenomes displayed the lowest taxa abundance (Figure 2). No significant differences (α = 0.05) in Shannon diverstiy (Figure 2) nor Pielou’s evenness (Supplementary Figure 4) were observed, though Dry Biocrust metagenomes, followed by Active Cell fraction metagenomes, were qualitatively observed to have higher Shannon diversity (Figure 2B) and Pielou’s evenness (Supplementary Figure 4). Additionally, Fraction (*R*^2^ = 0.398, Pr(>F) = 0.0009) significantly explained the variation in community composition; although non-significant, a trending effect was noted between Fraction and Timepoint (*R*^2^ = 0.065, Pr(>F) = 0.0859).

**FIGURE 3:**
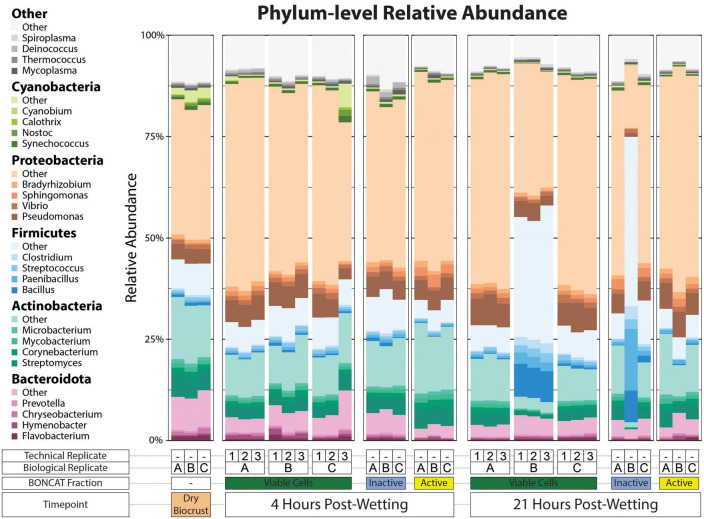
Phylum-level relative abundance of kraken2/bracken taxonomy output. The top five most abundant phyla across the dataset are plotted as five different color pallets and the top four genera of each of these phyla are filled with various shades of the phylum color pallet. Other top genera are filled with shades of gray. A key for the metagenome sample type is included below the *x*-axis.

All metagenomes were generally dominated by Proteobacteria, Actinobacteria, Bacteroidota, and Firmicutes regardless of Fraction or Timepoint (Figure 3). Cyanobacteria qualitatively showed a larger relative abundance in the Dry Biocrust metagenomes compared to the cell fractions. *Pseudomonas*, *Vibrio*, and *Sphingomonas* were among the most abundant genera of Proteobacteria. Among the Actinobacteria, *Streptomyces* and *Corynebacterium* were the major genera, while *Bacillus* and *Paenibacillus* were dominant among the Firmicutes. These genera were found across most metagenomes (Figure 3).

**FIGURE 4:**
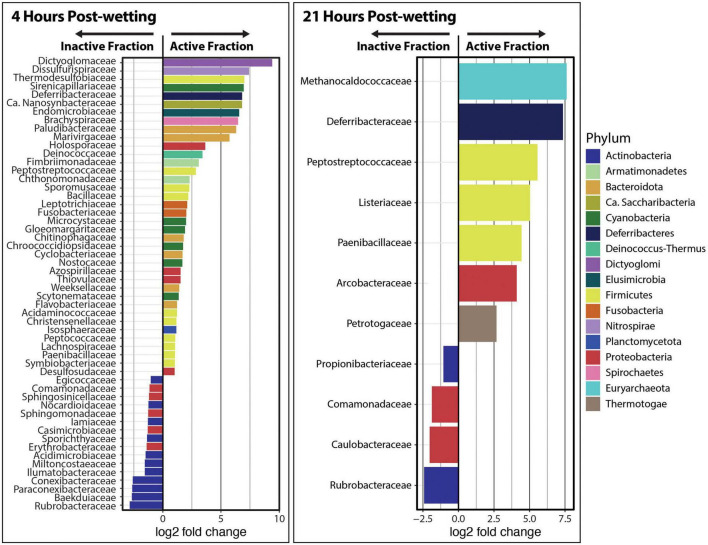
Differential abundance analysis of kraken2/bracken taxa at the family level at 4 and 21 hrs after biocrust wetting. Only significantly different families between the Inactive Cell and Active Cell fractions are plotted (α = 0.05). A positive log2 fold change value indicates higher abundance in the Active Cell fraction while a negative log2 fold change value represents a higher abundance in the Inactive Cell fraction. Barplot colors represent the phylum in which each family belongs.

“DESeq2” analyses were performed at the phylum and family levels in order to detect differentially abundant taxonomic groups between the Inactive Cell and Active Cell fractions. We found that Dictyoglomi, Deinococcus-Thermus, Armatamonadetes, Ignavibacteriae were significantly enriched (α = 0.05) in the Active Cell fraction at 4 hrs after biocrust wetting, while Euryarchaeota, Actinobacteria, and ‘Candidatus’ Thermoplasmatota were enriched in the Inactive Cell fraction (Supplementary Figure 5). In total, 38 families belonging to 14 phyla were detected at a larger abundance in the Active Cell fraction at 4 hrs compared to the Inactive Cell fraction (Figure 4). Most of these families (*n* = 34) belonged to phyla that were not statistically enriched in the Active Cell fraction at the phylum-level. In contrast, a smaller number of families (*n* = 16) – solely from the Proteobacteria and Actinobacteria phyla – were enriched in the Inactive Cell fraction at 4 h. No phyla were found at a significantly higher abundance in either the Inactive Cell or Active Cell fractions at 21 hrs post-wetting; however, seven families – belonging to five different phyla – were significantly enriched in the Active Cell fraction. Similar to trends observed at 4 h, a smaller number of families (four) were enriched in the Inactive Cell fraction, and these families belong to the Proteobacteria and Actinobacteria (Figure 4).

### Metagenome gene composition

**FIGURE 5:**
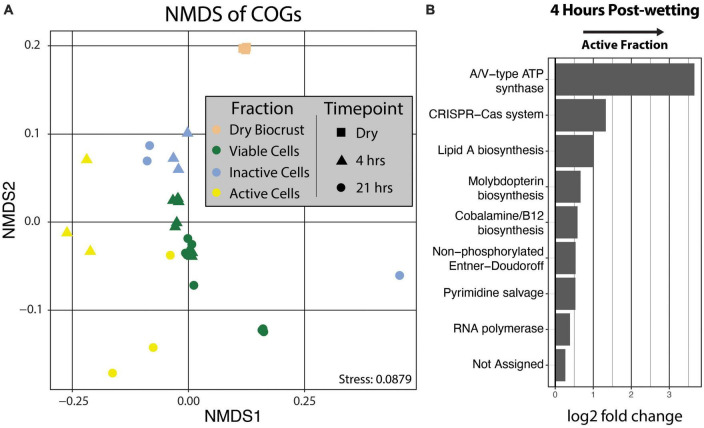
**(A)** Non-metric multidimensional scaling of COG abundances using Bray–Curtis distances. PERMANOVA analyses demonstrated significant grouping by Fraction (Pr(>F) = 0.0009) and by the combined effect of Fraction by Timepoint (Pr(>F) = 0.0019). Fraction is designated by point color and Timepoint is represented by point shape. **(B)** Differential abundance analysis of COG Pathways at 4 hrs post-wetting. COG Pathways were only significantly enriched in the 4 hrs Active Cell fraction (α = 0.05), and therefore 21 hrs post-wetting was not plotted. A positive log2 fold change value indicates higher abundance in the Active Cell fraction.

**FIGURE 6:**
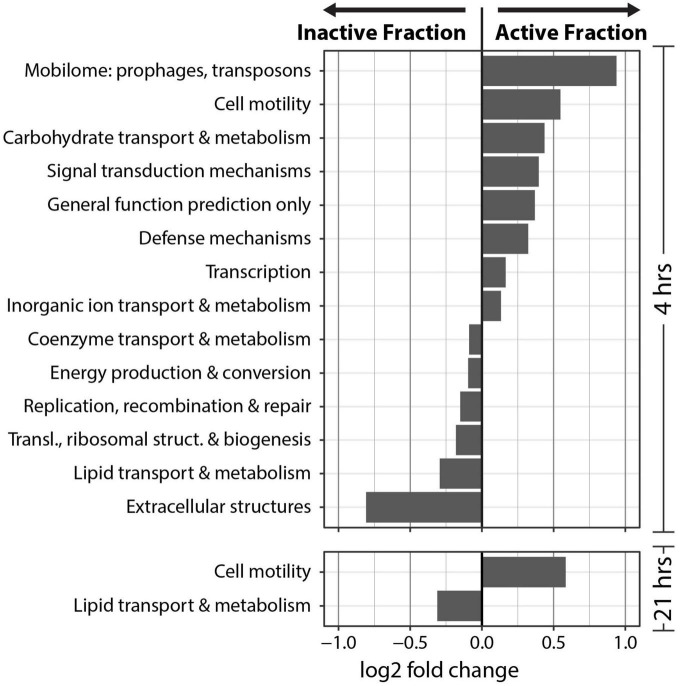
Differential abundance analysis of COG categories at 4 and 21 hrs post-wetting. A positive log2 fold change value indicates higher abundance in the Active Cell fraction. Only COG categories with a significant (α = 0.05) differential abundance were plotted.

Clusters of Orthologous Genes (COG) counts were extracted from IMG/M annotations of the metagenomes to estimate the differences in the genetic potential of the Active Cell and Inactive Cell metagenomes. PERMANOVA tests on the Bray-Curtis dissimilarities calculated from COG abundances demonstrated that Fraction (*R*^2^ = 0.43, Pr(>F) = 0.0009), and to a smaller degree the combined effect of Fraction by Timepoint (*R*^2^ = 0.11, Pr(>F) = 0.0019), explained the composition of the annotated COGs across samples (Figure 5A). Additionally, “DESeq2” analyses were performed at the “COG Category,” “COG Pathway,” and “COG ID” levels to determine the genetic features that differed between the Inactive Cell and Active Cell fractions. A total of eight COG Pathways were significantly more abundant in the Active Cell fraction at 4 hrs after wetting (Figure 5B), while no COG Pathways were differentially abundant at 21 h. No COG Pathways were significantly more abundant in the Inactive Cell fraction at either Timepoint. More broadly, eight COG Categories were enriched in the Active Cell fractions and six enriched in the Inactive Cell fraction at 4 h, while only two COG Categories differ between the Active Cell and Inactive Cell fractions at 21 hrs (Figure 6). A large number of individual COGs were significantly enriched in the Active Cell (4 h: 53 COGs, 21 h: 9 COGs) and Inactive Cell (4 h: 13 COGs, 21 h: 1 COG) fractions. Though these differentially abundant COGs span a wide range of COG Categories, many are related to carbohydrate transport and metabolism, as well as coenzyme transport and metabolism and secondary metabolite biosynthesis (Supplementary Tables 3, 4).

### Variation across biological and technical replicates

Generally, the trends in phyla-level taxonomic relative abundance within Cell Fraction type and two Timepoints were consistent across the three biological replicates (Figure 3); however, we observe that one biological replicate had a much larger relative abundance of Firmicutes at 21 hrs in both the Viable Cell and Inactive Cell fractions compared to the other two biological replicates. Our study design included technical replicates within the Viable Cell fractions at 4 and 21 hrs that allowed us to assess variability deriving from the method for this cell fraction. Here, in the Viable Cell fraction, phylum-level taxonomic relative abundances were largely consistent across technical replicates (Figure 3). Beta-dispersion analyses (Supplementary Figure 6) measuring the distances to group centroids in the NMDS space revealed that both taxonomic and COG within-group beta-dispersion were statistically largest in the Inactive Cell fraction at 21 hrs (taxonomic beta-dispersion: 0.45 ± 0.11, COG beta-dispersion: 0.25 ± 0.06). Taxonomic and COG within-group beta-dispersion was statistically similar among all other groups aside from Dry Biocrust metagenomes (taxonomic beta-dispersion: 0.020 ± 0.002, COG beta-dispersion: 0.003 ± 0.001) which had the smallest within-group beta-dispersion. Group dispersion increased across time for both taxonomic and COG data (Supplementary Figure 6).

## Discussion

### Reproducibility of BONCAT-FACS-Seq to probe active microbes in biocrusts

BONCAT-FACS-Seq is a powerful approach to probe the physiology of microbial cells and to assess the active microbial community function (Hatzenpichler and Orphan, 2015; Hatzenpichler et al., 2020). Here, we report the first insight on the microbial diversity of the active and inactive fractions of a microbial community from intact cores of a biological soil crust. We find that BONCAT-FACS-Seq identified distinct subsets of the extractible microbial community and effectively distinguished translationally active cells from inactive cells. The active component of the microbial community displayed less taxonomic diversity compared to the inactive fraction, suggesting that only a limited group of organisms initially respond to soil wetting while a larger fraction of the microbial diversity remains dormant.

BONCAT-FACS-Seq revealed nuanced differences in the taxonomic composition of the active and inactive fractions of the biocrust community after the wetting event. All metagenome samples, including the Dry Biocrusts, were dominated by Proteobacteria, Actinobacteria, Firmicutes, and Bacteroidota (Figure 3) – taxa which have previously been reported to be dominant in many other biocrust communities (Angel and Conrad, 2013; Karaoz et al., 2018; Van Goethem et al., 2019). Notably, we did not detect a repeatable bloom of Firmicutes at the phyla level, which has been observed previously in similar biocrusts as early as 9 hrs after wetting (Karaoz et al., 2018; Van Goethem et al., 2019). It is possible this particular biocrust community does not consistently undergo a bloom of Firmicutes, or it may undergo such a bloom later on in the wetting cycle, as evidenced by a large increase in the relative abundance of Firmicutes in the Viable Cell fraction of one biological replicate at 21 h. At the family level, we found 11 families of Firmicutes were enriched in the Active Cell fraction at both 4 and 21 hrs after wetting (even in the two samples that did not exhibit a clear bloom of Firmicutes, i.e., Biological Replicates A and C). We note that for the Firmicutes there is a disconnect between activity revealed by BONCAT and presumed activity from changes in relative abundance and previous knowledge. Here, one would expect to find a bloom of Firmicutes in the Active Cell fraction which is not what we observe. We suggest that these Firmicutes could have utilized using an alternate source of methionine in place of HPG – possibly from organics released at the onset of the wet-up event (Karaoz et al., 2018) – which would explain why we did not reliably observe them in the active fraction.

Additionally, we note that filamentous microorganisms such as Cyanobacteria are not reliably observed in our data, despite their importance in biocrust communities. Flow cytometry-based cell sorting constrains our ability to dependably sort large filamentous microorganisms, and therefore hinders the power to consistently identify whether these organisms are active or inactive. Previous reports have already characterized the activity of cyanobacteria in early successional biocrusts (Rajeev et al., 2013; Swenson et al., 2018), and although we do see evidence that number of families within the cyanobacteria are enriched in the Active Cell fraction at 4 h, we focus on the non-cyanobacterial component of the biocrust community.

We observed increasing variability in the biological signal with time which resulted in an attenuation of the discriminatory power of BONCAT-FACS-Seq over time after the addition of HPG. Beta-dispersion of both the taxonomic and functional data is significantly highest at 21 hrs (Supplementary Figure 6). Similarly, less taxa (Figure 4) and COGs (Figure 5B) are significantly different between the Active Cell and Inactive Cell fractions at 21 hrs compared to 4 hrs after wetting. This increased variation with time can be attributed to the fact that the BONCAT signal (i.e., HPG incorporation into biomass) is an accumulation of the physiological responses from the point of HPG addition across time up until destructive sampling, and does not represent the physiology of cells at a specific moment in time. This may suggest that microbial community responses to wetting are stochastic and follow increasingly unique paths over time. Additionally, more controlled studies are needed to fully evaluate how HPG might affect cellular metabolism. A recent publication showed that *Escherichia coli* metabolism was modified when growing on HPG (Steward et al., 2020). Although difficult to extrapolate from results obtained from a lab organism to an entire soil community *in situ*, we recommend based on this evidence and our study that BONCAT be used to provide a snap-shot of translationally active organisms, constraining the incubation time to not allow cells to grow and drift from their initial metabolic status. Such conditions would correspond to less than a few hours in soil, according to measurements of soil bacteria growth rates (Caro et al., 2023).

### Multidimensional shifts in the taxonomic composition of a biocrust community

Using BONCAT-FACS-Seq we successfully distinguished the diversity of the translationally active and inactive components of a biocrust undergoing a wet-up event. At the phyla level, the most abundant taxa were relatively invariable between the active and inactive components and across time. One notable exception was the Actinobacteria – a dominant bacterial phylum that was enriched here in the Inactive Cell fraction at 4 hrs after wetting. The Actinobacteria are known to be abundant in dry biocrusts and decrease in relative abundance after a wetting event (Angel and Conrad, 2013; Van Goethem et al., 2019; Baubin et al., 2022). Additionally, Actinobacteria are commonly reported in higher abundance in dry soils compared to wet soils (Goodfellow and Williams, 1983; Alekhina et al., 2001; Bachar et al., 2010; Nessner Kavamura et al., 2013; Niederberger et al., 2015), and in soils which experimentally received reduced water (Cruz-Martínez et al., 2009; Bouskill et al., 2013). At the family level, only families belonging to the Actinobacteria and Proteobacteria are statistically enriched in the Inactive Cell fraction at either 4 or 21 h. As Karaoz et al. (2018) noted an increase in abundance of Proteobacteria at 25.5 hrs after a wetting event in similar crusts, and we do not to observe active Proteobacterial families up to 21 hrs after wetting, we suggest Proteobacteria may undergo a slow response to wetting. Together these suggest the Actinobacteria and Proteobacteria members may remain inactive during very wet soil conditions (or early after a rain event) and their activity may be restricted to more xeric points in the hydration-desiccation cycle, which may hint at their particular niches in biocrust communities.

Among the families significantly enriched in the Active Cell fraction (Figure 4), many (11/42, 26%) belong to the Firmicutes – a phylum which, as noted earlier, was not statistically more abundant in the Active Cell fraction when comparing at the phylum-level. While previous studies have demonstrated that the Firmicutes bloom as early as 9 hrs after biocrust wetting (Angel and Conrad, 2013; Karaoz et al., 2018; Swenson et al., 2018; Van Goethem et al., 2019), we detected 10 families of Firmicutes enriched in the Active Cell fraction at 4 hrs following soil wetting. Among these, the *Bacillaceae*, *Paenibacillaceae*, *Peptostreptococcaceae* have been found in many biocrust communities (Liu et al., 2017; Maier et al., 2018; Aanderud et al., 2019; You et al., 2021) and are known to respond strongly to biocrust wetting (Karaoz et al., 2018; Van Goethem et al., 2019). These taxa are involved in important ecological processes such as being targets for viral predation [i.e., *Bacillaceae* (Van Goethem et al., 2019)] or N fixation [i.e., *Paenibacillaceae* (De Vos et al., 2011)]. Other families (e.g., *Sporomusaceae*, *Acidaminacoccaceae*, *Christensenellaceae*, *Peptococcaceae*, *Lachnospiraceae*, and *Symbiobacteriaceae*) are not well described in biocrust communities. Given their enrichment in the Active Cell fraction of the community, these may play important roles in community functioning and should be investigated further.

Additionally, we identify other families outside of the Firmicutes that are enriched in the Active Cell fractions. Many of these families, or in some cases subtaxa within them, have previously been noted in biocrust microbial communities (Table 1) and may hint at their importance biocrust ecology and function. Among these, the *Chitinophagaceae* have been described in many biocrust communities (Kuske et al., 2012; Angel and Conrad, 2013; Maier et al., 2018; Weber et al., 2018; Aanderud et al., 2019; Miralles et al., 2020; Glaser et al., 2022; Zhang et al., 2022) and seem to play important roles in the ecology and nutrient turnover in biocrusts. Members of the *Chitinophagaceae* are thought to be important in metabolizing carbohydrates (Aanderud et al., 2019) and degrading cyanobacterial-derived exopolymeric substances (Kuske et al., 2012). For example, the recently described *Candidatus Cyanoraptor togatus* is capable of predating Cyanobacteria within biocrust communities (Bethany et al., 2022), clearly demonstrating its’ importance in biocrust communities. In addition to these, numerous other families remain undescribed in biocrust communities and should be further investigated to illuminate their roles in biocrust community dynamics and function.

**TABLE 1:** Families, excluding those from the Firmicutes, found to be enriched in the active fraction of our biocrust community which have previously been described in other biocrust communities.

Taxa	References	Location	Note
*Dictyloglomeacea*	Blay et al., 2017	Intermountain West, USA	Found in minor abundance
*Deferribacteraceae*	Blay et al., 2017	Intermountain West, USA	Found in minor abundance
*Deinococcaceae*	Fisher et al., 2020; Pombubpa et al., 2020; Meier et al., 2021; Nevins et al., 2021; You et al., 2021; Baubin et al., 2022; Štovícek and Gillor, 2022	Idaho, USA; Florida, USA; Mojave Desert, USA; Negev Desert, Israel	Finds Deinococcus-Thermus phylum only in the “inactive” component of community (Baubin et al., 2022)
*Fimbriimonadaceae*	Couradeau et al., 2019a; García-Carmona et al., 2022	Alicante, Spain; Chihuahuan Desert, USA; Great Basin Desert, USA	Described genus *Fimbriimonas*
*Chthonomonadaceae*	Couradeau et al., 2019a	Chihuahuan Desert, USA; Great Basin Desert, USA	Described genus *Chthonomonas*
*Chitinophagaceae*	Kuske et al., 2012; Maier et al., 2018; Weber et al., 2018; Aanderud et al., 2019; Miralles et al., 2020; Glaser et al., 2022; Zhang et al., 2022	Great Basin Desert, USA; Northern Cape Province, South Africa; Various Mesic Forests, Germany; Tabernas Desert, Spain; Shaanxi Province, China	
*Azospirillaceae*	Miralles et al., 2020	Tabernas Desert, Spain	One OTU found in biocrust; Also on isolate found in desert soils (Li et al., 2021)
*Flavobacteriaceae*	Maier et al., 2018; Weber et al., 2018; Cania et al., 2020; Miralles et al., 2021	Tabernas Desert, Spain; Northern Cape Province, South Africa; Brandenburg, Germany	More often found in bare arid soils (Weber et al., 2018), but also very early successional biocrusts (Weber et al., 2018; Cania et al., 2020)
*Isosphaeraceae*	Miralles et al., 2020	Tabernas Desert, Spain	Found in early successional biocrust (Miralles et al., 2020), but also bare arid soil (Glaser et al., 2022)

### Differences in the potential functioning of the active and inactive components of a biocrust community

In order to understand the potential functions that are important to the response to biocrust wetting, we compared COG annotations between the active and inactive metagenomes. We find evidence to support recent reports (Van Goethem et al., 2019; Bethany et al., 2022) underscoring the importance of predation as a key ecological process in the early wet-up stage of biocrust hydration. Viral predation in biocrusts, including via temperate prophages, is known to exert control on Firmicutes population dynamics after a wetting event (Van Goethem et al., 2019). Furthermore, we found the CRISPR-Cas COG pathway to be significantly enriched in the active fraction at 4 h, specifically pointing toward active defense from viral predation among early responding bacteria. In addition to phage, as noted earlier, we observed an enrichment of *Chitinophagaceae* in the active fraction at 4 h, of which at least one member has been shown to predate on cyanobacteria in biocrust communities (Bethany et al., 2022). COGs assigned to “Defense mechanisms” were significantly more abundant in the Active Cell fraction at 4 hrs which would be expected if predation – by phage or by members of the *Chitinophagaceae* – is occurring in our biocrust community. Together, an abundance of mobilome related COGs, the CRISPR-Cas system COG Pathway, and COGs related more broadly to “Defense mechanisms” in the active fraction at 4 hrs after wetting further supports the idea that biocrust wetting quickly induces the predation of bacteria in biocrust communities, which has been noted before (Van Goethem et al., 2019; Bethany et al., 2022).

Notably, we find COG Categories related to achieving active population growth more abundant in the Inactive Cell fraction compared to the active fraction. These include COG Categories such as “Energy production and conversion,” “Replication, recombination, and repair,” “Translation, ribosomal structure, and biogenesis.” This may suggest that nutrient recycling and scavenging are important traits in the early response to biocrust wetting. For instance, “Carbohydrate transport and metabolism” and “Inorganic ion transport and metabolism” are more abundant in the active fraction and may indicate that early responsive organisms are relying on metabolizing existing resources in the biocrust environment, which is consistent with studies that observed high rates of labile C use after wetting in arid soils (Austin et al., 2004; Saetre and Stark, 2005). This may reflect the importance of predation whereby nutrients are released into the soil matrix from lysed cells, and/or the preference of early responding organisms to take advantage of easily accessible, labile compounds. The bioavailability of labile compounds for early responders to metabolize is likely very high, since there is a rapid release of metabolites into the soil matrix after wetting (Swenson et al., 2018). Nutrient recycling and scavenging may be particularly important life-history traits of the microorganisms that are able to be sorted by FACS; namely, microbes that are easily detachable from the soil matrix, as well as non-filamentous and planktonic cells. Interestingly, we observe an enrichment in COGs assigned to “Cell motility” in the Active Cell metagenomes at both 4 and 21 hrs after biocrust wetting, suggesting the importance of motility in this fraction of the microbial community.

## Conclusion

We confirmed that BONCAT-FACS-Seq is a novel, robust method that enables the identification of active microbes, and their genetic features, from intact soil communities under natural conditions. We speculate that when the incubation period is kept short (i.e., within a few hours) it ensures that HPG does not alter cell growth but is used to snapshot the activity of the community. Based on BONCAT probing, we find that many Actinobacteria and Proteobacteria members remain inactive through 21 hrs following wetting, while the Chitinophagaceae and some families within the Firmicutes are active and may play important roles in the community. According to COG abundances, early responder metagenomes are enriched in defense mechanisms, mobilome related COGs, and cell motility which could reflect the importance of active predation after wetting. Additionally, these metagenomes have higher abundances of COGs related to carbohydrate transport and metabolism and inorganic ion transport and metabolism, which may point toward a preference for the active organisms to utilize easily accessible and labile nutrients shortly after soil wetting. It remains unclear when, or if, the taxa that remained inactive through 21 hrs following the rain event would resuscitate, and under what environmental and biological conditions this would occur. Future studies should investigate to what degree the cumulative activation of microorganisms across successive hydration and desiccation cycles would recapitulate the total biodiversity measured in a biocrust community.

## Data availability statement

The raw sequencing data presented in the study are deposited in the NCBI SRA repository (https://www.ncbi.nlm.nih.gov/sra), accession number PRJNA938738. R code and associated data can be found at: https://github.com/RVTrexler/UT_Biocrust_BONCAT_Study/.

## Author contributions

EC, MV, TN, and RM conceptualized and designed the study. EC, MV, DG, and NN executed the microcosm manipulations, sampling, and sample processing. RT, EC, and MV performed the data processing and bioinformatics and drafted the manuscript. All authors contributed to the data analysis and interpretation and provided the insight toward revising the manuscript.

## Footnotes

1
https://gold.jgi.doe.gov/
