# Supplementary material for: BONCAT-FACS-Seq reveals the active fraction of a biocrust community undergoing a wet-up event
Source: Front Microbiol. 2023 Jun 26;14:1176751. doi: 10.3389/fmicb.2023.1176751 (PMC10330726; doi:10.3389/fmicb.2023.1176751)
Supplement: Supplementary file 1 [file Data_Sheet_1.docx]

Supplementary Material

BONCAT-FACS-Seq reveals the active fraction of a biocrust community undergoing a wet-up event

Ryan V. Trexler, Marc W. Van Goethem, Danielle Goudeau, Nandita Nath, Rex R. Malmstrom, Trent R. Northen, Estelle Couradeau*

*** Correspondence:** Corresponding Author: efc5279@psu.edu

# Supplementary Data

Supplementary Material should be uploaded separately on submission. Please include any supplementary data, figures and/or tables.

Supplementary material is not typeset so please ensure that all information is clearly presented, the appropriate caption is included in the file and not in the manuscript, and that the style conforms to the rest of the article.

# Supplementary Figures and Tables

For more information on Supplementary Material and for details on the different file types accepted, please see [here](https://www.frontiersin.org/guidelines/author-guidelines#supplementary-material).

**Supplementary File 1:** IMG/M Metagenome accessions.

**Supplementary File 2:** IMG/M Metagenome data and statistics

**Supplementary File 3:** Differentially abundant COGs at 4 hrs post-wetting

**Supplementary File 4:** Differentially abundant COGs at 21 hrs post-wetting

## Supplementary Figures

**
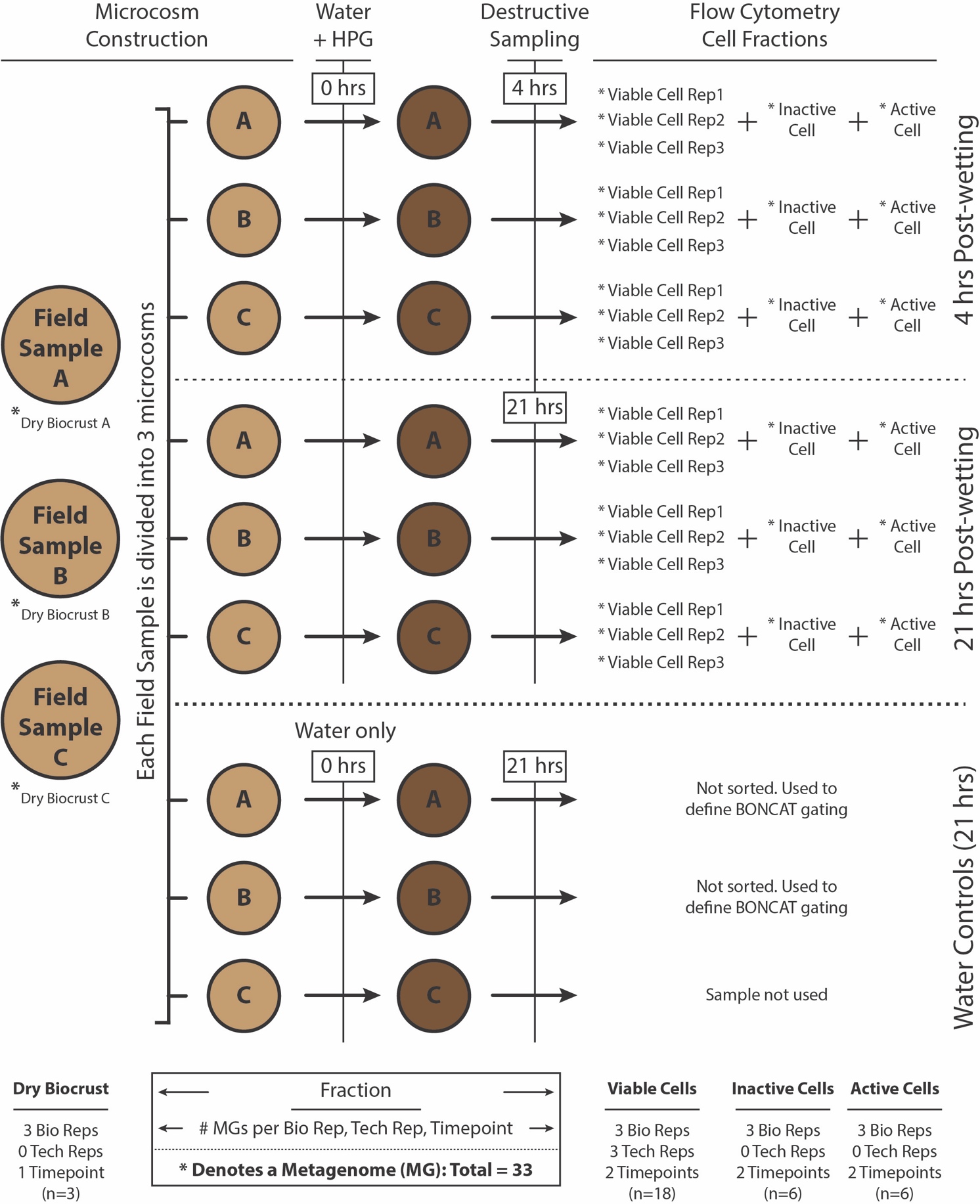
**

**Supplementary Figure 1:** Experimental design showing field samples, microcosms, sampling events, and flow cytometry fractions. The three Field Samples of a biocrust community were each subsampled into three separate microcosms (Bio Reps), resulting in a total of 9 microcosms. Six microcosms were watered with L-homopropargylglycine (HPG) in order to simulate a rain event. The remaining three microcosms received only sterile water, and served as Water Control samples. Three of the HPG-receiving microcosms were destructively harvested at 4 hrs after wetting, while the other three microcosms that received HPG were destructively harvest at 21 hrs following wetting. The three microcosms that received only water were harvested at 21 hrs, though only two were used in the BONCAT gating step. For a detailed description of the gating strategy, see Figure 1 and Supplementary Figure 2. In total, three technical replicates of Viable Cell fractions, and one technical replicate of Active and Inactive Cell fractions were collected for each of the three biological replicates at each of the two timepoints. Shotgun metagenomes (MG) were generated from the dry Field Samples (Dry Biocrust) before subsampling, and the three cell fractions collected following cell sorting – Viable Cell, Inactive Cell, and Active Cell. Stars indicate that a shotgun metagenome was prepared from the sample. No metagenomes were collected from the Water Controls.

**Supplementary Figure 2:** Overview of the flow cytometry-based cell sorting used to generate the Viable Cell, Inactive Cell, and Active Cell fractions from the incubated microcosms. A. SYTO59 fluorescence was used to collect samples of intact, viable cells (Viable Cell fraction), discriminating these particles against non-stained particles. B, E. Two microcosms that received only water were used to identify the background FAM Picolyl azide dye fluorescence. Fluorescence above this background (giving a false positive rate of > 0.05%) represented active cells (Active Cell gate). The Inactive Cell gate was drawn at least a half an order of magnitude lower in FAM Picolyl azide dye fluorescence. C, F. When sorting, cells within the Viable Cell gate and Inactive Cell gate were assigned to the Inactive Cell fraction, while cells falling within the Viable Cell and Active Cell gate were assigned to the Active Cell fraction. Cell sorting was performed on microcosms receiving HPG and harvested at either 4 or 21 hrs following the wetting event.


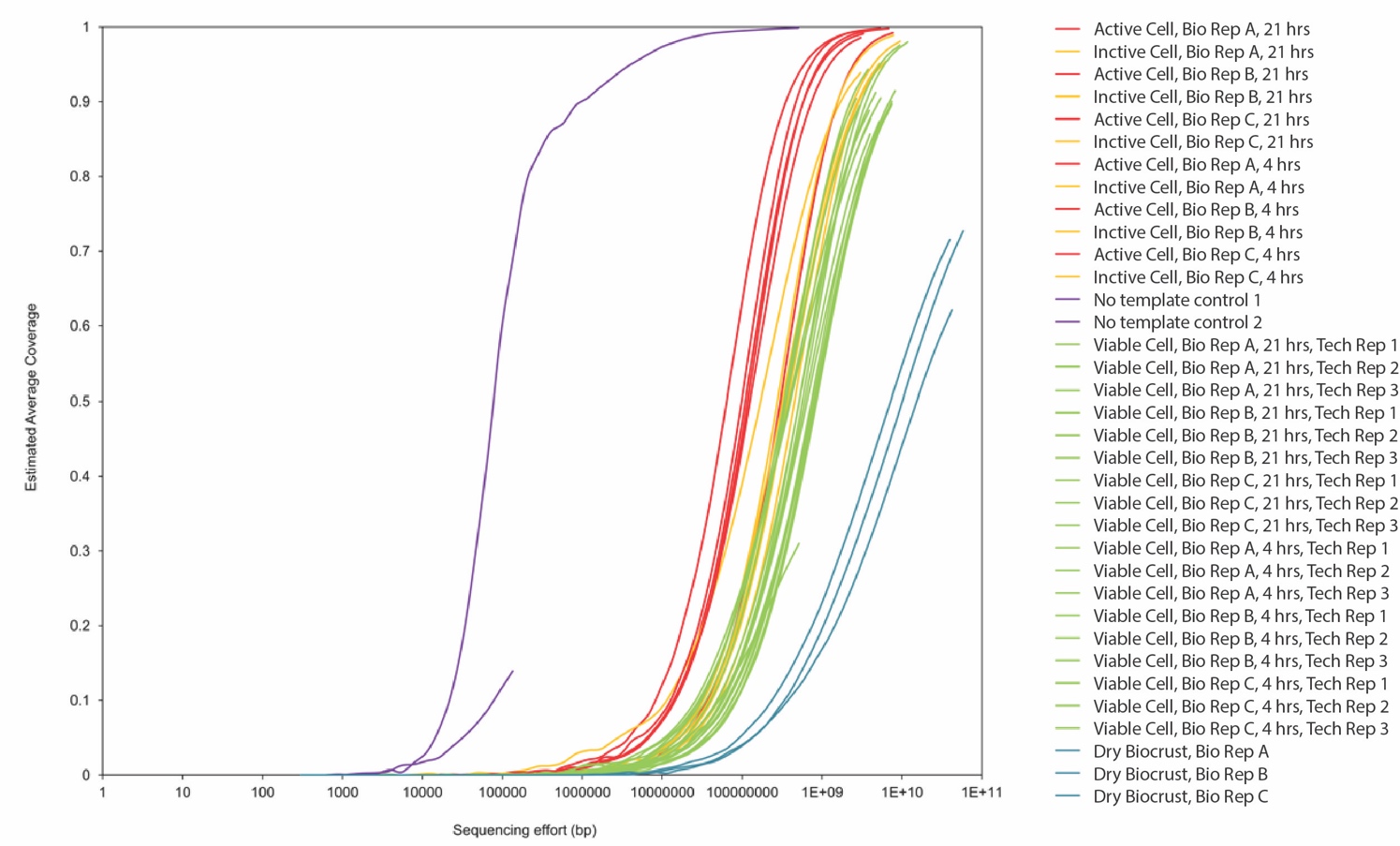


**Supplementary Figure 3:** Non-pareil curves estimating the completeness of the metagenomes. Blue lines represent the Dry Biocrust metagenomes (n=3). Viable Cell fractions are shown in green (n=18), Active Cell fractions in red (n=6), and Inactive Cell fractions in yellow (n=6). Two no-template controls are in purple.


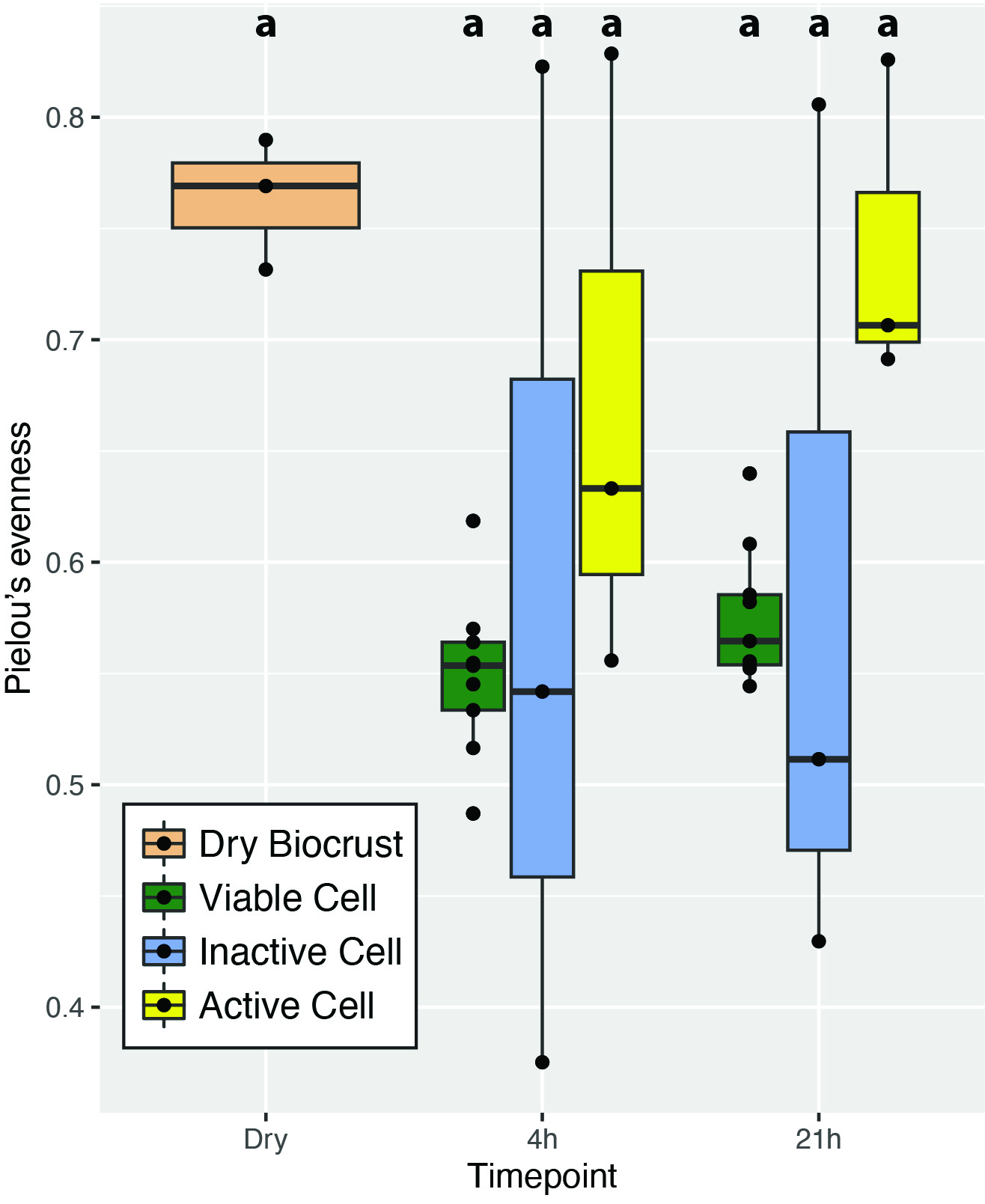


**Supplementary Figure 4:** Boxplots of Pielou’s evenness calculated from the kraken2/bracken taxonomy output. No significant differences (α = 0.05) were detected between any treatment.


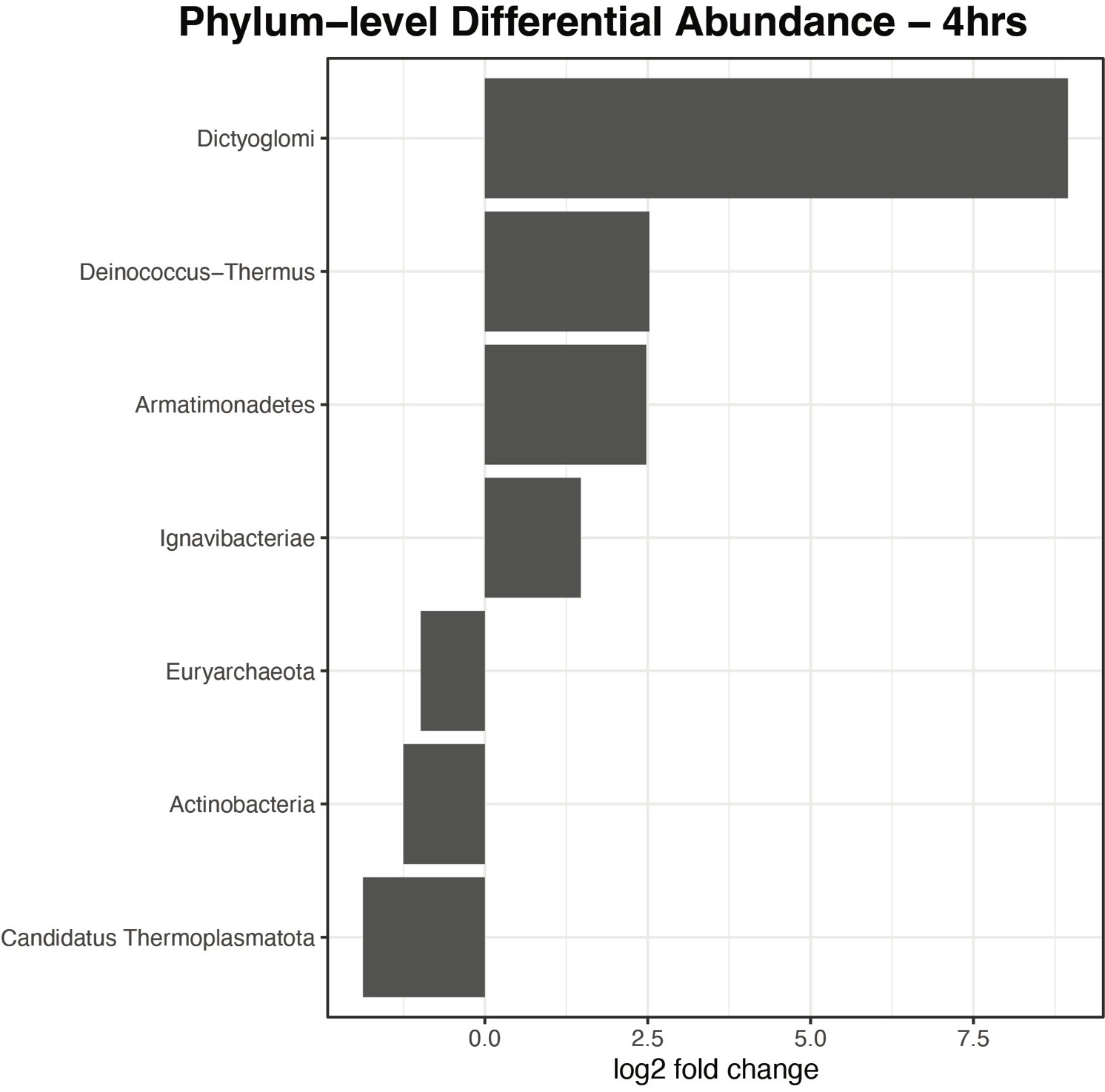


**Supplementary Figure 5:** Differential abundance analysis of kraken2/bracken taxa at the phylum level at 4 hrs after biocrust wetting. Only significantly different phyla between the Inactive Cell and Active Cell fractions are plotted (α = 0.05). A positive log2 fold change value indicates higher abundance in the Active Cell fraction while a negative log2 fold change value represents a higher abundance in the Inactive Cell fraction. No significantly different phyla were found at 21 hrs post-wetting, and thus, not plotted.

**Supplementary Figure 6.** Boxplots of beta-dispersion estimates calculated using the Euclidian distance from the group centroid in NMDS space for both taxonomic (A) and COG (B) data. Differences in beta-dispersion among groups (‘abc’) were tested using two-way ANOVAs with post hoc Tukey’s HSD tests (α = 0.05).
